# 第三方骨髓来源间充质干细胞治疗异基因造血干细胞移植后难治性迟发性出血性膀胱炎20例临床研究

**DOI:** 10.3760/cma.j.issn.0253-2727.2022.06.008

**Published:** 2022-06

**Authors:** 珂 赵, 芬 黄, 小湧 陈, 媛 昌, 娜 许, 鹏程 史, 慧 刘, 竞 孙, 鹏 项, 启发 刘, 志平 范

**Affiliations:** 1 南方医科大学南方医院血液科，广州 510515 Department of Hematology, Nanfang Hospital, Southern Medical University, Gangzhou 510515, China; 2 中山大学干细胞与组织工程研究中心，广州 510120 Center for Stem Cell Biology and Tissue Engineering, Sun Yat-Sen University, Guangzhou 510080, China

**Keywords:** 难治性迟发性出血性膀胱炎, 异基因造血干细胞移植, 间充质干细胞, Refractory late-onset hemorrhagic cystitis, Allogeneic hematopoietic stem cell transplantation, Mesenchymal stromal cell

## Abstract

**目的:**

观察第三方骨髓来源间充质干细胞（MSC）治疗异基因造血干细胞移植（allo-HSCT）后难治性迟发性出血性膀胱炎（LOHC）的疗效和安全性。

**方法:**

回顾性分析2018年7月至2020年6月allo-HSCT后发生难治性LOHC 20例患者在常规治疗基础上联合第三方骨髓来源MSC治疗。MSC以每次1×10^6^/kg、每周1次输注，直至症状改善或连用4次无效停用。在MSC治疗前及治疗后第8周应用定量PCR法检测患者尿液标本中BK病毒（BKV）、JC病毒（JCV）、巨细胞病毒（CMV）。

**结果:**

①20例难治性LOHC患者中，男15例、女5例，中位年龄35（15～56）岁；急性淋巴细胞白血病（ALL）5例，急性髓系白血病（AML）9例，骨髓增生异常综合征（MDS）5例，母细胞性浆细胞样树突细胞瘤（BPDCN）1例；HLA全相合移植4例，HLA不全相合移植16例。②MSC输注中位次数为3（2～8）次。17例患者获得完全缓解，1例获得部分缓解，总缓解率为90.0％。移植后中位随访397.5（39～937）d，13例存活、7例死亡，死亡原因包括急性GVHD 1例、感染5例、血栓性微血管病（TMA）1例。③MSC输注后第8周尿BKV-DNA及CMV-DNA拷贝数较治疗前显著降低（11342.1×10^8^拷贝/L对5.2×10^8^拷贝/L，*P*＝0.016；3170.0×10^4^拷贝/L对0.2×10^4^拷贝/L，*P*＝0.006），而JCV-DNA与治疗前相比无明显改变（*P*＝0.106）。④未发生MSC输注相关不良反应。

**结论:**

第三方骨髓来源MSC对allo-HSCT后难治性LOHC具有显著疗效且安全性良好。

## Abstract (en)

**Objective:**

To examine the efficacy and safety of third-party bone marrow-derived mesenchymal stem cells (MSCs) in the treatment of refractory delayed hemorrhagic cystitis (LOHC) after allogeneic hematopoietic stem cell transplantation (allo-HSCT).

**Methods:**

Twenty patients with refractory LOHC received conventional therapy combined with MSCs obtained from third-party donors' bone marrow (BM). MSCs were given intravenously at a dose of 1 × 10^6^ cells/kg once weekly until either the symptoms improved or no changes in LOHC were seen after continuous infusion four times. BK viruria (BKV)-DNA, JC viruria (JCV)-DNA, and CMV-DNA were detected by real-time quantitative PCR before and 8 weeks after the MSCs infusion.

**Results:**

① Of the 20 patients with refractory LOHC, 15 were males, and 5 were females, and the median age was 35 (15–56) years. There were 5 cases of acute lymphoblastic leukemia (ALL), 9 cases of acute myeloid leukemia (AML), 5 cases of myelodysplastic syndrome (MDS), and 1 case of maternal plasma cell like dendritic cell tumor (BPDCN). There were 4 cases of HLA identical transplantation and 16 cases of HLA incomplete transplantation. ②The median number of MSC infusions for each patient was 3 (range: 2–8). Seventeen patients achieved complete response, and one had a partial response after treatment. The overall response rate was 90%. Over a median follow-up period of 397.5 days (range 39–937 days) post-transplantations, 13 patients survived, and 7 died. The causes of death included aGVHD (1 case), infections (5 cases), and TMA (1 case). ③The copy numbers of BKV-DNA and CMV-DNA in urine in the 8th week after MSCs infusion were significantly lower than those observed before treatment (11342.1×10^8^ copies/L *vs* 5.2×10^8^ copies/L, *P*＝0.016; 3170.0×10^4^ copies/L *vs* 0.2×10^4^ copies/L, *P*＝0.006, respectively), while JCV-DNA did not significantly differ when compared to before treatment (*P*＝0.106). ④ No adverse reactions related to MSC infusion occurred in any of the 20 patients.

**Conclusion:**

Third-party bone marrow-derived MSC has significant efficacy and good safety in the treatment of refractory LOHC after allogeneic HSCT.

迟发性出血性膀胱炎（LOHC）是异基因造血干细胞移植（allo-HSCT）后的严重并发症之一，其发生率为5％～49％[1]–[2]。LOHC的发病机制尚未阐明，可能与急性移植物抗宿主病（aGVHD）及病毒感染有关[3]。LOHC的治疗主要以支持治疗为主，包括水化、碱化、利尿、血小板输注等[4]。轻、中度LOHC通过支持治疗多可治愈，但重度LOHC多持续数月，常规治疗效果不佳，最终可导致尿路梗阻、肾功能衰竭甚至危及生命。间充质干细胞（MSC）因具有炎性和免疫调节、组织修复等功能[5]–[6]，用于LOHC治疗取得了一定疗效[7]–[10]。近年来，我们采用第三方骨髓来源MSC治疗20例allo-HSCT后难治性LOHC患者，旨在探索难治性LOHC的新疗法。

## 病例与方法

1. 病例：本回顾性研究纳入2018年7月至2020年6月在我院行allo-HSCT后发生难治性LOHC且未接受过MSC治疗的20例患者。本研究经南方医科大学南方医院伦理委员会批准，在clinicaltrials.gov网站登记（NCT02241018）。所有患者、供者或监护人均签订知情同意书。

2. 移植方案：所有患者采用白消安（Bu）为基础的预处理方案。HLA全相合同胞移植采用环孢素A（CsA）+甲氨蝶呤（MTX）+霉酚酸酯（MMF）方案预防GVHD；HLA不全相合及无关供者移植采用CsA+MTX+MMF+抗胸腺细胞球蛋白（ATG）方案[11]。aGVHD诊断分级采用Mount Sinai aGVHD国际联盟（MAGIC）制定的标准[12]。

3. LOHC预防与治疗：LOHC预防：预处理期间给予水化、碱化尿液、利尿，补液量2.5～3.0 L·m^−2^·d^−1^，尿pH维持在7.0～8.0，保持尿量在3000 ml/d以上。预处理方案包含环磷酰胺（Cy）时，在Cy使用前快速静脉给予每日总量25％的美司钠，余量分3次（每8 h 1次）维持24 h泵入，末次Cy给药后24～48 h仍予每日总量75％的美司钠匀速持续泵入。LOHC治疗：发生LOHC的所有患者均接受水化、碱化尿液、短时间抗生素及输注血小板等支持治疗。症状无改善或合并病毒感染者，予减少免疫抑制剂、更昔洛韦或膦甲酸钠、西多福韦等抗病毒治疗；合并GVHD者，予抗GVHD治疗。对于重度LOHC患者，可给予高压氧及外科干预等治疗。

4. LOHC诊断标准、分度及疗效评价：出血性膀胱炎（HC）诊断标准根据文献[13]，即肉眼或镜下血尿伴尿频、尿急、尿痛，且除外泌尿系肿瘤、感染等引起的血尿。HC按发生时间分为早发性HC（发生于预处理期间或预处理结束3 d内）和LOHC（发生于预处理结束3 d后）[14]。出血程度分级根据Droller标准[15]分为Ⅰ度（镜下血尿）、Ⅱ度（肉眼血尿）、Ⅲ度（肉眼血尿伴小血凝块）和Ⅳ度（肉眼血尿伴血凝块阻塞尿道，引起肾功能衰竭）。Ⅰ、Ⅱ度为轻度，Ⅲ、Ⅳ度为重度。保守治疗1周无效的LOHC定义为难治性LOHC。疗效评价标准：①完全缓解（CR）：膀胱刺激症状消失，无镜下血尿且长期随访无复发；②部分缓解（PR）：指膀胱刺激症状较前好转（排尿次数减少至少25％或尿痛、排尿困难缓解）或血尿程度至少下降1度；③无效（NR）：肉眼血尿无减轻或进行性加重。总有效率（OR）指CR+PR患者在所有患者中的占比。

5. MSC来源、扩增及鉴定：MSC均来源于无关供者HLA不相合的第三方BM。按我们文献描述方法采集BM、分离和培养扩增MSC[16]。在无菌条件下采集供者骨髓，离心后收集单个核细胞。按5000个细胞/cm^2^悬浮于人MSC生长培养基中贴壁培养，待细胞接近80％融合时消化传代。采用流式细胞术（FACS）检测MSC表面抗原表达，鉴定MSC向成骨和成脂细胞分化潜能。取培养第4、5代MSC供治疗用。

6. MSC治疗：所有患者均在原有治疗基础上接受MSC治疗。MSC以每次1×10^6^/kg经外周静脉输注（30 min），每周1次，直至症状改善或连用4次无效而停用。在MSC输注后48 h内，密切监测患者的生命体征。

7. 尿病毒panel检测：采用QT-PCR方法在MSC治疗前及治疗后第8周检测患者尿中巨细胞病毒（CMV）、EB病毒（EBV）、BK病毒（BKV）、JC病毒（JCV）及腺病毒定量。

8. 随访：分别在MSC输注前及输注后+1 d、+2 d、+7 d、+14 d、+21 d、+28 d、+42 d、+56 d记录患者生命体征、症状、尿量、尿色、尿常规、血常规、肾功能等。中位随访397.5（39～937）d，随访截止日期为2021年7月30日。

9. 统计学处理：应用SPSS 21.0软件进行统计学分析。定量资料均数比较采用配对或两独立样本*t*检验，定性资料比较采用卡方检验。*P*<0.05为差异有统计学意义。

## 结果

1. 病例一般资料（表1）：20例难治性LOHC患者中，男15例、女5例，中位年龄35（15～56）岁。急性淋巴细胞白血病（ALL）5例，急性髓系白血病（AML）9例，骨髓增生异常综合征（MDS）5例，母细胞性浆细胞样树突细胞瘤（BPDCN）1例。20例患者中，HLA全相合移植4例、HLA不全相合移植16例。19例为相关供者移植（同胞供者9例、父母或子女供者10例）、1例为无关供者移植。外周血造血干细胞（PBSC）移植4例、PBSC与骨髓混合移植16例。预处理方案：16例采用BU-CY（白消安+环磷酰胺）方案，4例采用BF方案（白消安+氟达拉滨）。GVHD预防方案：4例采用CsA+MTX+MMF方案、16例采用CsA+MTX+MMF+ATG方案。在难治性LOHC发生时，20例患者中有11例存在aGVHD（Ⅱ度4例、Ⅲ度2例、Ⅳ度5例）；11例存在病毒血症（CMV血症10例，CMV及EBV血症并存1例）；全部20例患者的尿液标本中均检出病毒（BKV 7例，BKV+JCV 7例，BKV+CMV 2例，BKV+JCV+CMV 1例，BKV+CMV+腺病毒2例，CMV+BKV+JCV+EBV 1例）。

**表1:** 20例异基因造血干细胞移植后难治性LOHC患者的一般资料

例号	性别	年龄（岁）	原发病	移植前疾病状态	移植预处理方案	aGVHD分度	病毒血症	尿病毒感染
1	男	15	AML	CR	BU-CY	Ⅱ	CMV	BKV、JCV
2	男	24	MDS	NR	BU-CY	Ⅳ	CMV	BKV
3	女	55	AML	CR	BU-CY	Ⅲ	CMV	BKV
4	男	22	ALL	CR	BU-CY	Ⅳ	CMV	BKV、JCV
5	男	30	MDS	NR	BU-CY	Ⅱ	NA	BKV
6	男	17	ALL	CR	BU-CY	Ⅲ	CMV	BKV
7	女	50	MDS	NR	BU-CY	无	NA	BKV
8	女	30	AML	CR	BF	无	NA	BKV
9	女	23	BPDCN	CR	BU-CY	无	NA	BKV
10	男	48	MDS	NR	BU-CY	无	NA	BKV、JCV
11	男	40	AML	CR	BF	无	CMV	BKV、JCV、CMV、EBV
12	男	56	ALL	CR	BF	无	NA	BKV、JCV
13	男	46	AML	PR	BU-CY	Ⅳ	CMV	BKV、JCV
14	男	44	AML	CR	BU-CY	Ⅱ	CMV	BKV、JCV
15	男	56	MDS	NR	BU-CY	Ⅳ	CMV、EBV	BKV、JCV、CMV
16	男	30	AML	CR	BU-CY	无	NA	BKV、JCV
17	男	20	AML	CR	BU-CY	无	CMV	BKV、CMV、腺病毒
18	男	50	ALL	CR	BF	Ⅱ	NA	BKV、CMV
19	女	21	ALL	CR	BU-CY	无	NA	BKV、CMV
20	男	28	AML	CR	BU-CY	Ⅳ	CMV	BKV、CMV、腺病毒

注：LOHC：迟发性出血性膀胱炎；AML：急性髓系白血病；ALL：急性淋巴细胞白血病；MDS：骨髓增生异常综合征；BPDCN：母细胞性浆细胞样树突细胞瘤；aGVHD：急性移植物抗宿主病；CR：完全缓解；PR：部分缓解；NR：未缓解；BU：白消安；CY：环磷酰胺；CMV：巨细胞病毒；EBV：EB病毒；BKV：BK病毒；JCV：JC病毒。NA：未检出

2. 20例难治性LOHC患者的临床表现、治疗及转归（表2）：LOHC中位发生时间为移植后31（21～91）d。Ⅱ度LOHC 1例（5％），Ⅲ度13例（65％），Ⅳ度6例（30％）。

**表2:** 20例异基因造血干细胞移植后难治性LOHC患者的临床表现、治疗及随访结果

例号	移植后LOHC发生时间（d）	LOHC分度	LOHC治疗		LOHC疗效	随访时间（d）	随访结果
			MSC治疗次数	其他			
1	+26	Ⅲ	8	免疫抑制剂、膦甲酸钠	CR	590	生存
2	+30	Ⅲ	8	免疫抑制剂、膦甲酸钠	CR	919	生存
3	+28	Ⅲ	2	免疫抑制剂、膦甲酸钠	CR	153	死亡
4	+63	Ⅲ	7	免疫抑制剂、西多福韦、膦甲酸钠	CR	795	生存
5	+21	Ⅳ	8	免疫抑制剂、西多福韦、膀胱镜血块清除	CR	806	生存
6	+50	Ⅲ	3	免疫抑制剂、西多福韦、膦甲酸钠	CR	269	死亡
7	+24	Ⅱ	5	西多福韦	CR	339	生存
8	+32	Ⅲ	4	西多福韦、高压氧	CR	383	生存
9	+24	Ⅳ	4	西多福韦	CR	937	生存
10	+30	Ⅲ	3	西多福韦、膀胱动脉栓塞	CR	487	生存
11	+71	Ⅳ	2	西多福韦、更昔洛韦	CR	347	死亡
12	+91	Ⅲ	2	西多福韦	CR	187	死亡
13	+45	Ⅲ	3	免疫抑制剂、膦甲酸钠	CR	735	生存
14	+26	Ⅲ	2	免疫抑制剂、膦甲酸钠	CR	448	生存
15	+54	Ⅲ	2	免疫抑制剂、西多福韦、膦甲酸钠	PR	71	死亡
16	+42	Ⅲ	6	西多福韦、高压氧	CR	617	生存
17	+34	Ⅳ	5	西多福韦、膦甲酸钠、膀胱镜血块清除、高压氧	CR	612	生存
18	+25	Ⅳ	3	免疫抑制剂、西多福韦、高压氧、膀胱镜血块清除	NR	39	死亡
19	+27	Ⅳ	2	西多福韦、膀胱镜血块清除	NR	105	死亡
20	+44	Ⅲ	3	免疫抑制剂、西多福韦、膦甲酸钠	CR	634	生存

注：LOHC：迟发性出血性膀胱炎；MSC：间充质干细胞；CR：完全缓解；PR：部分缓解；NR：未缓解

11例合并有aGVHD的难治性LOHC患者应用糖皮质激素等免疫抑制剂治疗：甲泼尼龙单药4例，甲泼尼龙+CsA+MMF+芦可替尼5例，甲泼尼龙+CsA+MMF+抗CD25单克隆抗体2例。在开始MSC治疗前，11例合并有aGVHD的难治性LOHC患者均为NR。20例血、尿标本检出病毒的患者均接受抗病毒治疗，其中单用西多福韦9例、单用膦甲酸钠5例、西多福韦+膦甲酸钠5例、西多福韦+更昔洛韦1例。在开始MSC治疗前，20例血、尿标本检出病毒患者的病毒检测均未转阴。另外，4例患者接受高压氧治疗、11例留置尿管膀胱冲洗、5例外科干预（膀胱镜下血块清除4例、膀胱动脉栓塞1例）。经上述治疗后，20例难治性LOHC患者在开始MSC治疗前LOHC均未获得缓解。

20例患者共接受79例次MSC输注，中位输注次数为3（2～8）次。首次输注MSC的中位时间是在发生难治性LOHC后10（5～43）d。17例患者获得CR，1例PR，2例NR，OR率为90.0％。尿路刺激症状中位消失时间为MSC治疗后12（5～30）d，肉眼血尿中位消失时间为MSC治疗后16（6～58）d，镜下血尿中位消失时间为MSC治疗后20（9～95）d。14例Ⅱ、Ⅲ度难治性LOHC患者均获得CR（CR率为100％），而6例Ⅳ度LOHC患者中3例获得CR、1例获得PR（CR率为50％，OR率为66.7％）。MSC对Ⅱ、Ⅲ度难治性LOHC治疗的CR率和OR率均高于Ⅳ度患者（*P*＝0.004，*P*＝0.023）。3例患者死亡前LOHC未愈，其他患者截止至随访日期均获得CR。

11例合并aGVHD的患者经糖皮质激素等免疫抑制剂及MSC治疗，10例患者的aGVHD获得CR，1例NR。且在MSC输注后没有新发aGVHD出现。

3. MSC的不良反应：没有患者发生任何MSC输注的急性不良反应。除1例发生EBV相关移植后淋巴细胞增殖性疾病（PTLD）外，没有出现其他的继发肿瘤。

4. MSC治疗前后尿病毒变化：20例难治性LOHC患者在MSC治疗前尿标本均检出病毒，其中20例（100％）检出BKV、9例检出JCV、6例检出CMV、2例检出腺病毒、1例检出EBV。13例患者检出2种以上病毒。MSC输注后8周尿BKV-DNA和CMV-DNA拷贝数显著低于治疗前（*P*＝0.016，*P*＝0.006），而JCV-DNA拷贝数与治疗前相比差异无统计学意义（*P*＝0.106），详见表3。

**表3:** 异基因造血干细胞移植后难治性LOHC患者MSC治疗前及治疗后8周尿病毒检测结果［*M*（范围）］

组别	BKV-DNA（20例，×10^8^/L）	CMV-DNA（6例，×10^4^/L）	JCV-DNA（9例，×10^8^/L）
治疗前	11342.1（60.0～78800.0）	3170.0（1000.0～5520.0）	13204.6（173.0～58100.0）
治疗后	5.2（1.0～12.0）	0.2（0.0～0.6）	1310.4（0.0～6230.0）

*t*值	2.650	4.499	1.823
*P*值	0.016	0.006	0.106

注：LOHC：迟发性出血性膀胱炎；MSC：间充质干细胞；BKV：BK病毒；CMV：巨细胞病毒；EBV：EB病毒；JCV：JC病毒

5. 随访结果：移植后中位随访467.5（39～937）d。20例患者中13例存活，7例患者死于其他移植相关并发症（感染5例，Ⅳ度aGVHD、血栓性微血管病各1例）。

## 讨论

LOHC是allo-HSCT后的常见并发症，严重者可导致尿路梗阻、肾功能衰竭，直接影响移植成功率和患者生存率[17]。目前，LOHC的治疗方式取决于疾病的严重程度及进展速度，通常以保守治疗为主，包括水化、碱化尿液、利尿、血小板输注等。对于难治性LOHC患者，基于其发病可能与病毒感染和GVHD相关，可加用西多福韦等抗病毒治疗。Simone等[18]应用西多福韦治疗57例BKV相关出血性膀胱炎（HC）患者，38例（67％）获得CR，7例（12％）获得PR。Mo等[19]研究发现：83例经抗病毒治疗未获得CR的LOHC患者给予糖皮质激素治疗，CR率77.1％。

MSC是存在于多种组织中的一种成体干细胞，因具有向多系细胞分化、免疫和炎性调节、修复组织等功能在防治GVHD及抗感染等领域受到广泛的研究与关注[5]–[6],[20]。在2007年，Ringdén等[21]首先报道7例allo-HSCT后重度LOHC患者接受BM来源MSC治疗后5例获得缓解。随后，Wang等[9]应用第三方脐带来源MSC治疗7例allo-HSCT后LOHC患者，3例患者在MSC输注后肉眼血尿消失。Tong等[8]进一步将研究聚焦在BKV相关的重度LOHC患儿，13例患儿接受脐带血来源MSC治疗后均获得缓解，总有效率高达100％。在本回顾性小样本研究中，20例难治性LOHC在常规治疗基础上，采用第三方BM来源MSC治疗，17例患者获得CR，1例获得PR，OR率为90％。这一结果与文献报道的MSC治疗重度LOHC疗效相当。这表明MSC不仅对重度LOHC有显著疗效，而且对常规治疗无效的难治性LOHC也有显著疗效。

MSC治疗难治性LOHC的机制尚不明确。有研究认为MSC可通过先天性和获得性免疫改善aGVHD这一LOHC的高致病因素，其作用机制包括：抑制T细胞增殖与功能，诱导调节性T细胞产生，抑制B细胞的增殖、活化与抗体分泌等[6], [8]–[9], [22]。此外，我们前期研究表明MSC亦有抗感染作用，除有直接抗病原微生物作用外[20]，在体内因可促进移植后免疫重建而间接发挥抗病原微生物作用[16]。在本研究中，我们对MSC治疗前、治疗后8周患者尿BKV、CMV拷贝数检测，发现MSC治疗后难治性LOHC患者尿BKV、CMV拷贝数较治疗前显著下降，同时aGVHD也得到有效控制。本组病例结果初步显示MSC对allo-HSCT后难治性LOHC治疗有效，其机制可能与其抗病毒和调控aGVHD作用有关。

## References

[1] Bogdanovic G, Priftakis P, Giraud G (2004). Association between a high BK virus load in urine samples of patients with graft-versus-host disease and development of hemorrhagic cystitis after hematopoietic stem cell transplantation[J]. J Clin Microbiol.

[2] Leung AY, Suen CK, Lie AK (2001). Quantification of polyoma BK viruria in hemorrhagic cystitis complicating bone marrow transplantation[J]. Blood.

[3] Leandro de Padua Silva, Patah PA, Saliba RM (2010). Hemorrhagic cystitis after allogeneic hematopoietic stem cell transplants is the complex result of BK virus infection, preparative regimen intensity and donor type[J]. Haematologica.

[4] Harkensee C, Vasdev N, Gennery AR (2008). Prevention and management of BK-virus associated haemorrhagic cystitis in children following haematopoietic stem cell transplantation--a systematic review and evidence-based guidance for clinical management[J]. Br J Haematol.

[5] Uccelli A, Moretta L, Pistoia V (2008). Mesenchymal stem cells in health and disease[J]. Nat Rev Immunol.

[6] Zhao K, Liu Q (2016). The clinical application of mesenchymal stromal cells in hematopoietic stem cell transplantation[J]. J Hematol Oncol.

[7] Ringden O, Le Blanc K (2011). Mesenchymal stem cells for treatment of acute and chronic graft-versus-host disease, tissue toxicity and hemorrhages[J]. Best Pract Res Clin Haematol.

[8] Tong J, Liu HL, Zheng CC (2020). Effects and long-term follow-up of using umbilical cord blood-derived mesenchymal stromal cells in pediatric patients with severe BK virus-associated late-onset hemorrhagic cystitis after unrelated cord blood transplantation[J]. Pediatr Transplant.

[9] Wang Y, Chen F, Gu B (2015). Mesenchymal stromal cells as an adjuvant treatment for severe late-onset hemorrhagic cystitis after allogeneic hematopoietic stem cell transplantation[J]. Acta Haematol.

[10] 邱 坤银, 廖 雄宇, 郭 淑仪 (2018). 间充质干细胞治疗儿童难治性迟发性出血性膀胱炎疗效和安全性的临床研究[J]. 中国实验血液学杂志.

[11] Wang Y, Liu QF, Lin R (2021). Optimizing antithymocyte globulin dosing in haploidentical hematopoietic cell transplantation: long-term follow-up of a multicenter, randomized controlled trial[J]. Sci Bull (Beijing).

[12] Harris AC, Young R, Devine S (2016). International, multicenter standardization of acute graft-versus-host disease clinical data collection: a report from the Mount Sinai Acute GVHD International Consortium[J]. Biol Blood Marrow Transplant.

[13] Gorczynska E, Turkiewicz D, Rybka K (2005). Incidence, clinical outcome, and management of virus-induced hemorrhagic cystitis in children and adolescents after allogeneic hematopoietic cell transplantation[J]. Biol Blood Marrow Transplant.

[14] Russell SJ, Vowels MR, Vale T (1994). Haemorrhagic cystitis in paediatric bone marrow transplant patients: an association with infective agents, GVHD and prior cyclophosphamide[J]. Bone Marrow Transplant.

[15] Droller MJ, Saral R, Santos G (1982). Prevention of cyclophosphamide-induced hemorrhagic cystitis[J]. Urology.

[16] Zhao K, Lou R, Huang F (2015). Immunomodulation effects of mesenchymal stromal cells on acute graft-versus-host disease after hematopoietic stem cell transplantation[J]. Biol Blood Marrow Transplant.

[17] Cesaro S, Facchin C, Tridello G (2008). A prospective study of BK-virus-associated haemorrhagic cystitis in paediatric patients undergoing allogeneic haematopoietic stem cell transplantation[J]. Bone Marrow Transplant.

[18] Cesaro S, Hirsch HH, Faraci M (2009). Cidofovir for BK virus-associated hemorrhagic cystitis: a retrospective study[J]. Clin Infect Dis.

[19] Mo XD, Zhang XH, Xu LP (2018). Treatment of late-onset hemorrhagic cystitis after allogeneic hematopoietic stem cell transplantation: the role of corticosteroids[J]. Ann Hematol.

[20] Qin AP, Lai DH, Liu QF (2017). Guanylate-binding protein 1 (GBP1) contributes to the immunity of human mesenchymal stromal cells against Toxoplasma gondii[J]. Proc Natl Acad Sci USA.

[21] Ringdén O, Uzunel M, Sundberg B (2007). Tissue repair using allogeneic mesenchymal stem cells for hemorrhagic cystitis, pneumomediastinum and perforated colon[J]. Leukemia.

[22] English K, French A, Wood KJ (2010). Mesenchymal stromal cells: facilitators of successful transplantation?[J]. Cell Stem Cell.

